# An Overview on the Rheology, Mechanical Properties, Durability, 3D Printing, and Microstructural Performance of Nanomaterials in Cementitious Composites

**DOI:** 10.3390/ma14112950

**Published:** 2021-05-30

**Authors:** Hongwei Song, Xinle Li

**Affiliations:** College of Civil Engineering, Dalian Minzu University, Dalian 116600, China; linxinle@dlnu.edu.cn

**Keywords:** cementitious composites, material characteristics, nanomaterials, mechanical properties, cement matrix

## Abstract

The most active research area is nanotechnology in cementitious composites, which has a wide range of applications and has achieved popularity over the last three decades. Nanoparticles (NPs) have emerged as possible materials to be used in the field of civil engineering. Previous research has concentrated on evaluating the effect of different NPs in cementitious materials to alter material characteristics. In order to provide a broad understanding of how nanomaterials (NMs) can be used, this paper critically evaluates previous research on the influence of rheology, mechanical properties, durability, 3D printing, and microstructural performance on cementitious materials. The flow properties of fresh cementitious composites can be measured using rheology and slump. Mechanical properties such as compressive, flexural, and split tensile strength reveal hardened properties. The necessary tests for determining a NM’s durability in concrete are shrinkage, pore structure and porosity, and permeability. The advent of modern 3D printing technologies is suitable for structural printing, such as contour crafting and binder jetting. Three-dimensional (3D) printing has opened up new avenues for the building and construction industry to become more digital. Regardless of the material science, a range of problems must be tackled, including developing smart cementitious composites suitable for 3D structural printing. According to the scanning electron microscopy results, the addition of NMs to cementitious materials results in a denser and improved microstructure with more hydration products. This paper provides valuable information and details about the rheology, mechanical properties, durability, 3D printing, and microstructural performance of cementitious materials with NMs and encourages further research.

## 1. Introduction

The rising impact of greenhouse gases has resulted in a severe issue of global warming, putting the atmosphere at risk of seasonal shifts, ice melting, and an increase in overall air and ocean temperatures. The CO_2_ level in the United States decreased in 2017 compared with 2016, owing to the transition from coal to natural gas, renewable energy sources, and other factors [1]. To halt global warming, human activities such as fossil fuel combustion, cement manufacturing, and deforestation must reduce CO_2_ emissions to zero. The planet will get hotter the longer it takes us to do so. The lack of progress toward decarbonization has sparked understandable concern about the climate crisis. This has sparked a renewed interest in technical climate interventions that include injecting substances into the stratosphere to cause the formation of highly reflective particles, which increase the reflection of sunlight to space. Since first proposed, such albedo modification schemes have been presented as a “Plan B” if the global economy failed to decarbonize, and this scenario has dominated much of the public image of albedo modification as a savior waiting in the wings to save the planet from catastrophic climate change caused by a failure to decarbonize [2]. The vast nanotechnology market and its growing use by every sector is also a source of concern because of the toxicological effects of nanomaterials (NMs) on human health and the environment. Since NMs are small, researchers have discovered evidence of NMs entering the human body through various routes, including ingestion, skin penetration, and inhalation. When these particular NMs enter the bloodstream, they can cause a variety of neurological disorders as well as some serious health issues. The increasing globalization of NMs is also harmful to the climate. The presence of NMs in the air can damage soil fertility, marine life, and uncontaminated water bodies and contribute to increasing environmental issues such as dust cloud formation and the stratospheric temperature. The physiochemical properties of NMs, such as size, surface area, shape, charge, aggregation, and so on, have an impact on their toxicity [1,3,4]. NMs can veer toward agricultural and water resources when exposed to the environment. NMs play an essential role in creating dusty clouds, and their existence in the Earth’s atmosphere may affect various ecological processes. Brown clouds include nano-sized carbon components as well as particulate matter resulting from the combustion of fossil fuels, the emission of soot-containing industrial fumes, and the combustion of plastic waste, among other things [1]. The effects of NMs in cementitious materials are close to the effects of micro-based materials. Many researchers have noticed pore refinement as well as improved material strength and durability. The main difference is that the material’s size is reduced to the nanoscale. Since micro-based materials have been shown to increase the strength and durability of cementitious materials, using nanoparticles in cementitious materials is likely to similarly provide further improvements in mechanical properties (MPs) [5].

**Figure 1:**
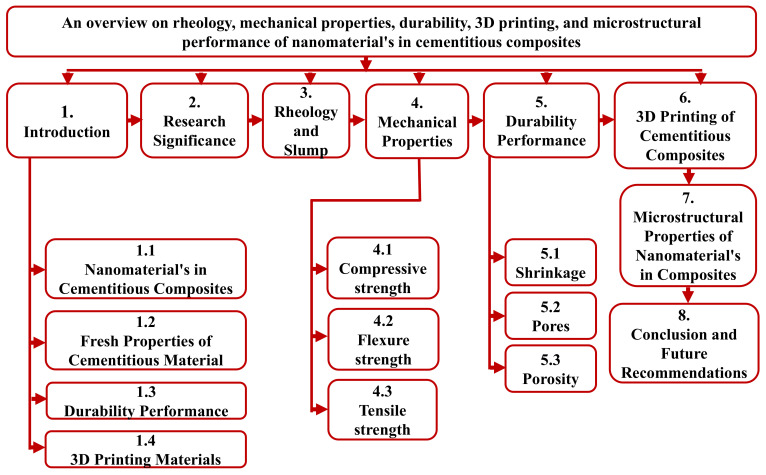
Outline of this study.

The use of NMs in cementitious materials has improved their characteristics significantly in recent years. The addition of NMs to cementitious materials is to monitor the behavior of the material and attain improved properties in terms of durability and mechanical performance [6]. On the other hand, carbon nanotubes (CNTs), nanographene oxide (NGO), nano alumina (NA), nano titania (NTI), and nano iron-oxide (NIO) have been used to achieve new properties like self-sensing, self-healing, and self-cleaning [7]. A NM’s addition to cementitious materials will considerably promote the rate of hydration, the electrical resistivity, and the delivery of more nucleation sites for calcium silicate hydrate (C-S-H) gel precipitation and growth. Because of the different hydration products, the mechanical and durability properties have improved [8]. The water demand grows as the maximum specific surface area (SSA) increases, and the matrix’s overall workability decreases. The use of superplasticizers (SPs) can increase the rheological and workability properties of printed concrete [9]. Various NMs, as shown in Figure 1, have been used to improve the performance of cementitious composites [10].

### 1.1. Nanomaterials in Cementitious Composites

The term ‘nanomaterial’ was coined in the early 1980s to describe a subdivision size range of 1 nm to 100 nm with a wide specific surface area and an emphasis on metallic nanoparticles (NPs), non-metallic NPs, carbon-based NPs, mineral NPs, and so on [11]. Nano-sized (NSd) materials elude the researcher’s attention due to their peculiar chemical and physical properties, such as properties of the surface, smaller size effects, and so on, that are not found in macroscopic materials [12]. NMs have revealed possible applications in the areas of defense engineering, chemical industries, computer engineering, aerospace technologies, biotechnology-related engineering, and pharmaceutical applications over the last five decades based on their extraordinary properties [13]. More reactivity is seen in 4 nm NPs, which have around 50% of their molecules near the outer region [14]. The high surface area behavior of nano-silica (NS) was observed to minimize the usable free water in mixtures of cement. The NSd silica and pozzolanic fly ash (FA) resulted in improved physical properties, MPs, and a dense microstructure in hardened concrete [15]. By accelerating the suspension of tri-calcium silicate (C_3_S) and forming improved bond media, NS provides nucleation sites for C-S-H [14]. As shown in Table 1, the addition of NMs usually results in pore alteration and, as a result, lesser permeability. However, integrating these NMs has other advantages, such as improved mechanical efficiency, self-cleaning, and thermal performance. The influence of several NM types in cementitious composites on their corresponding output is shown in Table 1.

Another way to achieve a similar nucleation effect is to introduce 0.3% of C-S-H seeds into the pore medium, which promotes the formation of C-S-H bunches in the pore medium in place of the C_3_S stages [24]. On the other hand, the improvement in the pore medium accelerates the initial strength of mixtures, which can be quantified through microstructural investigations [25]. The formation of a densified microstructure of concrete using zeolite and inorganic NPs has been demonstrated in a study [10]. The cost of NMs has decreased in recent years because of increased research in material science and technology, and supply has been improved for different applications in the field of construction [26]. Combination of numerous NPs in conventional cement, binary mixed cement, ternary mixed cement, and advanced types of cement, such as magnesium phosphate cement (MPC), calcium sulfoaluminate cement, and magnesium ammonium phosphate cement (MAPC), in preparing several categories of concrete, such as early strength concrete, fast-setting concrete, and high-performance concrete, is being investigated by a large number of enthusiastic researchers [27,28,29,30,31].

NS is a highly effective pozzolanic material with glassy particles 1000 times finer than those found in cement [32,33]. NS is used as an additive to increase the cementitious matrix’s performance, including porosity and mechanical strength. NS has a significant impact on hydration kinetics due to its high SSA, enhancing microstructure and promoting pozzolanic action. NS interacts with calcium hydroxide (Ca(OH)_2_) during hydration and serves as a nucleation site for the development of C-S-H gels [34,35,36]. However, a rise in the hydration phase because of its chemical reactivity or greater SSA has yet to be determined [37]. NTI is primarily employed as a photo-catalyst and a coating material. However, as research on photo-catalytic materials has progressed, NTI has found a promising application in the building materials and construction field, where it has proven to be helpful in the de-pollution and self-cleaning of concrete [38]. Furthermore, when used in cementitious materials, NTI imparts pozzolanic properties, thus releasing a tremendous quantity of heat during the immediate reactions [34]. By increasing the NTI material, early temperature peaks were observed. As NTI is added to the concrete, it reduces the flowability and the initial and final setting times [39]. NTI affects the flowability of self-compacting concrete (SCC) in the same way. It was also reported that adding NTI to SCC increases the yield stress and consistency while lowering segregation and bleeding [40]. Based on its morphology and chemical composition, nano clay (NC) is graded as bentonite, hectorite, montmorillonite, kaolinite, halloysite, or organically altered NC [41]. NC raises the risk of hydration and the degree of flocculation by forming a tight bond with the cementitious materials. NC’s water absorption property can decrease the quantity of free water in a solution, raising the viscosity and yield stress [42]. NC has been commonly used in SCC to achieve early age strength and reduce formwork strain. Various researchers have looked into the effects of NC, FA, and extra additive materials on the SCC and pressure difference on formwork. The lowest pressure was achieved by using NC with less than 1% binders [43,44,45]. Table 2 shows the physical properties and optimum dosage of several NMs as stated in the literature.

NA increases the pozzolanic activity, effectively utilizes Ca(OH)_2_, and accelerates the formation of silicate and aluminum stages, resulting in the development of high hydration heat [46,47]. The impact of two forms of NA (g-phase and a-phase) on cement paste and mortar hydration products was investigated [48]. According to the findings, the NA (g-phase) particles formed a cluster. The NA (a-phase) particles were evenly distributed throughout the cement matrix and improved hydration products in the form of C-S-H gel and calcium aluminate hydrate (CAH) salts. Graphene oxide (GO) is an engineered NM due to its high SSA and elastic modulus, and thus it has shown itself to be a sturdy constituent [49]. The self-sensing properties of graphene-based NMs have made them broadly used for detecting cracks and damage without external equipment [50]. Significant increases in compressive and flexure strength were observed due to the creation of the strong chemical and physical bond with the cement matrix, the size effect, and the filler effect [51,52,53]. However, nano graphene (NG) flakes have non-uniform dispersion in the matrix, restricting their wide application. Graphene-based sheets were found to be economical and have improved properties and strain sensing [54]. The rate of hydration improves as the SSA increases and the agglomeration of cement particles decreases. With a minimal particle size and a larger surface area, they agglomerate and may serve as nucleation sites [55,56].

CNTs are produced from graphene sheets rolled into tubes or cylinders. Single-walled CNTs (SWCNTs) and multi-walled CNTs (MWCNTs) are the two types of CNTs. The length of both types is around 1 mm, but the diameters are different [63]. CNTs are one-dimensional graphene cylinders with a large length-to-diameter ratio. CNTs have attracted a lot of attention because of their physical and chemical properties, and they are being used in composite materials, catalysts, and sensor systems [64,65]. CNTs may be used in cementitious materials, depending on their size and morphology. According to researchers, CNTs can be incorporated into cementitious materials as a filler to improve the microstructure and, consequently, the MPs. However, these improvements are contingent on the CNT’s length-to-diameter ratio and dispersion in the matrix [66]. Additionally, CNTs have a substantial effect on the hydration rate. The impact of hydration on a blended paste of cement with SWCNTs at a water–cement ratio (w/c) of 0.5 was investigated using a calorimeter examination, scanning electron microscopy (SEM), and thermo-gravimetric analysis (T-G-A). The SWCNTs served as a nucleation site for the C_3_S hydration product by entrapping free water and minimizing fluidity [67]. Thus, using NMs in cementitious composites is beneficial for improving the overall performance.

### 1.2. Fresh Properties of Cementitious Material

Fresh concrete is a highly concentrated suspension of solid aggregates in a viscous cement paste liquid. Cement paste is a non-uniform material made up of cement particles scattered in the water. Since the sizes of the particles in concrete vary from mm to mm, it has complicated properties in a fresh state [68,69,70]. The effect on the consolidation, casting, and production processes, which can affect the long-term hardened concrete’s strength, necessitates the characterization of fresh concrete properties. Honey combing and segregation concerns may be induced by inadequate mixing and workability. As a result, good rheological properties are needed for the fresh concrete during pumping (pouring) and casting. Pumpability, mobility, workability, consistency, compactness, and finishing ability have all been used to describe the fresh properties of concrete in the past [71]. However, methodological approaches focused on qualitative research had only that one value. The flow/spread table test and the slump test are the most well-known tests for evaluating the fresh properties of cementitious materials. However, a slight difference in procedures has different outcomes. These tests were found to have a higher sensitivity [72]. Therefore, procedures to evaluate fresh concrete’s properties using physical quantities had to be developed.

Rheology is a concept that refers to how a material deforms and flows when subjected to applied stresses and shear speeds. In 1920, Bingham coined the word “rheology”. Flow curves, which relate yield stress, shear rate, and plastic viscosity, better recognize fresh concrete’s flowability. Rheology parameters may be used to test these concrete properties in their fresh state quantitatively and objectively. Rheology is linked to fresh concrete properties such as compactness, flowability, and stability [73]. The rheological properties of fresh concrete are associated with pumping pressure, segregation, surface finish consistency, and formwork pressure [74]. It was also discovered that lowering the plastic viscosity could lower the formwork pressure while increasing the risk of segregation [75].

Similarly, as the yield tension rises, the pumping pressure of rigid concrete increases. An appropriate combination of rheological parameters is needed to attain the best surface finish without segregation and minimal pumping pressure [9]. Rheological metrics, rather than slump flow measurements, may be used to assess high-performance concrete’s workability [76]. The thixotropy of cement paste has been observed to minimize the formwork’s strain, and this built-up structural phenomenon is advantageous in multilayer casting [77,78]. From regular vibrated concrete to SCC, the effect of rheological parameters and flow rate on pumping pressure differs [79]. Several analytical tests have been used to connect fresh properties to calculated quantities. To simulate concrete flow, rheometer tests and various models have been presented. Rheometers are preferred because they have physical measurements that are linked to the concrete’s fundamental flow activity. For applications such as 3D printing and SCC, these values can be used to estimate the flowability of fresh concrete. Concrete modeling is used to predict the material’s flow behavior more accurately based on its structure, as it provides more precise values once completely formed [80]. Models based on previous compositions were found to affect NM composites with aggregate properties such as scale, surface texture, and shape [81]. Hence, the rheology and slump are the important parameters used to determine the flowability and workability of cementitious composites containing NMs.

### 1.3. Durability Performance

The durability of cementitious composites is determined by their ability to withstand harmful forces of the environment. The ease with which decaying ions and fluids penetrate cementitious composites, as well as the amount of time it takes for them to do so, determines the material’s durability. Because of their ultrafine nature, NMs can act as pore filler between cement grains, resulting in a dense microstructure and improved penetration resistance to the harmful material. Furthermore, the NMs’ pozzolanic reactivity allows them to react with Ca(OH)_2_, resulting in the formation of extra C-S-H and consequently an increase in the density of the microstructure of the composite [16]. At 28 days, NS with 0.3% content decreased the water penetration depth by 56%, while NS at 0.9% content marginally increased the depth of water penetration, indicating that NS at a higher dose has an agglomeration effect. Accordingly, about a 91% increment was observed with 0.9% NS content and exhibited more water infiltration than 0.3% content [82].

Similarly, at 3.8% incorporation, colloidal NS and the powder form of NS result in an 88.5% decrease in the water penetration depth compared with the control mixture, indicating pore refinement and less interconnected pores [35,61]. NS particles in the size range from 10 to 40 nm were found to increase the cement mortar’s resistance to abrasion and water permeability. In terms of the NS size effect, a size of 40 nm was found to be successful in increasing pozzolanic behavior, particle scattering, and packing density. MPs and the microstructure were influenced by agglomeration and dispersion of the smaller particles, i.e., 12 nm [83]. Resistance to the penetration of water in cementitious composites increases the protection of a steel reinforcement from chloride-produced corrosion by improving the resistance to the penetration of chloride ions [82,84]. Because of synergetic effects, blended cement concrete has a higher resistance to water penetration [85,86]. The same synergetic effect is seen when NS and rice husk ash (RHA) are mixed [87]. These techniques may also create Ca(OH)_2_ nucleation sites early in the hydration process, speeding up the C_3_S phase hydration [62,88,89,90,91]. Initial and later age strength and a substantial decrease in water permeability are observed due to the combined effect of NS and RHA, as this combination works against dilution [24]. Various initial studies concentrated primarily on the MPs for quick-setting and high early strength concrete. There appears to be a gap in the research and information on the microstructure, bond properties of the fiber mix, MPs, and resilience of quick-setting and high early strength concrete systems containing various fiber types and NMs [10]. Therefore, NMs play a key role in enhancing cementitious composites’ characteristics.

### 1.4. 3D Printing Materials

Charles Hull first implemented additive manufacturing (AM) technology, also known as 3D printing, in the stereolithography (SLA) process in 1986. Following that, it has attracted every person’s interest, from industrialists to at-home hobbyists [92]. This is primarily because of the ability for freeform design, complex geometries manufacturing, waste material minimization, mass customization, and fabrication speed [93]. Even though it has been nearly 30 years since the first audit of 3D printers in the manufacturing sector, several processing problems, such as void creation, anisotropic conduct, design software limitations, and the low resolution of printed parts, still exist in construction and manufacturing processes. Furthermore, rigid and static sections continue to plague AM technology. As a result, several ongoing studies aim to overcome the current obstacles, such as by combining smart materials with AM techniques [94,95]. From the standpoint of printer equipment and materials, it is clear that 3D printing technology has advanced significantly. Three-dimensional (3D) printers can now attain dimensional precision, surface roughness, and enhanced MPs for end-user products.

**Figure 2:**
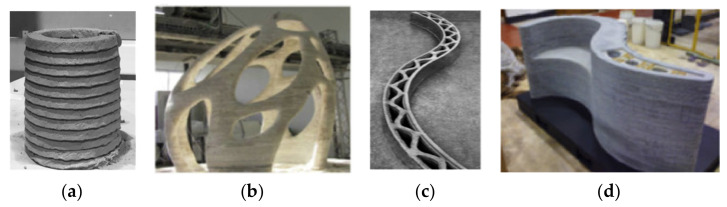
3D printing: (**a**) 3D printed object [106]; (**b**) D-Shape; (**c**) Contour crafting; (**d**) Concrete printing [98].

Furthermore, significant material advances have turned 3D printing technology into a medium for material science, allowing researchers and scientists to customize material deposition, anisotropic activity, and active sensing based on the environment [95,96]. Concrete printing differs from contour crafting in finishing and post-processing because of the ribbed finish production shown in Figure 2a [97]. Figure 2b–d depict the comparison of constructed components created using the various 3D structural printing techniques [98]. These accomplishments would pave the way to further 3D printing technology advancements. For example, in the medical field, the ‘Bio Pen’, which performs in situ 3D printing, is a very appealing and creative category of bio-printing that allows surgeons to deliver tissue engineering techniques at the time and place they need them [99,100]. The degree of flocculation and the hydration rate of fresh cement paste containing attapulgite clay were measured. The rheological parameters of 3D-printed cementitious materials were significantly affected by NC. With the addition of attapulgite, both the hysteresis loop curve and calorimeter tests revealed higher hydration and thixotropy rates [101]. NC improves the printed layers’ shape stability in 3D concrete printing [102]. When diutan gum and NC were compared in 3D concrete printing, it was discovered that NC offers good shape stability and reduces the time gap by acquiring early age strength without sacrificing flowability [103]. The influence of different contents of attapulgite clay on the rawness, microstructure, and printability of 3D cementitious mixtures containing a higher content of FA was investigated. Because of the dense microstructural development, the rheometer outcomes revealed a substantial enhancement in yield stress and apparent viscosity [104]. Furthermore, the degree of flocculation was high, suggesting that NC improves buildability and is well suited to 3D concrete printing. Similar results were found for the rheological behavior of 3D-printed composites containing NC [105]. In summary, nanomaterials are also useful in 3D printing for improving the performance of cementitious materials.

## 2. Significance of This Study

The existing research is summarized to enable researchers to learn about NMs’ applications in cementitious materials. This state-of-the-art review first introduces the effect of NMs on the characteristics of concrete in terms of the fresh state, the hardened state, durability, 3D printing, and microstructure. By examining the most recent research findings, this study aims to review and address the current literature with more than 250 articles on the effect of different types of NM incorporation on the overall behavior of cementitious materials in detail. The parameters include rheology, workability, compressive, tensile, and flexural strength, shrinkage, porosity, pore structure, 3D printing, and microstructure. This comprehensive overview of nanomaterials in cement-based materials is considered to provide a baseline for future research. The information included in this review would be helpful to those in learning about the use of NMs in cementitious materials. Researchers can take advantage of the summary presented in this manuscript of NMs’ entire performance and characteristics.

## 3. Rheology and Slump

**Figure 3:**
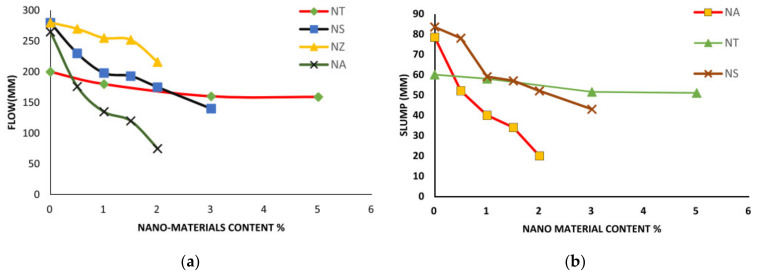
Impact of various NMs: (**a**) flowability; (**b**) slump [9].

Concrete is a construction material made up of cement, aggregates, mineral admixtures, water, and chemical admixtures in various sizes. Concrete’s rheology is primarily determined by the ingredients’ quality and their contacts. Numerous scholars have previously examined the impact of ingredients on concrete’s rheology. The rheology of cementitious nanocomposites is also influenced by other parameters, such as cement hydration and fineness, cement paste amount, temperature, the process of mixing, time of mixing, vibration, strain, and number of voids. This section explores and addresses these contributing factors [69,107]. Several studies examined the influence of NS, NTI, NA, NF, and Nano-ZnO (NZ) on mortar fluidity. The fluidity of mortars was found to be significantly reduced by NS, NF, and NA, while NZ and NTI had little impact (Figure 3a) [9]. In addition, for a slight rise in NA relative to NS, the slump value was suddenly decreased. However, in the case of the addition of NTI, the slump loss was marginal [38,108,109,110,111,112,113,114]. It has been claimed that concrete containing 15 nm size NA as a cement substitute loses its workability [109,112]. As a filler, NA adsorbs free water, which increases as the percentage of NA rises. In addition, the initial and final setting times were substantially reduced (Figure 3b) [9].

Various amounts of GO have been incorporated into cement paste. The GO blended paste’s rheological properties using the Bingham, Reformed Bingham, and Herschel Bulkley models were investigated [115]. The outcomes exhibited a significant rise in compressive and flexural strength but a considerable decrease in paste fluidity. The rheometer results showed that increasing the amount of GO in the cement paste improved the viscosity and yield stress. In the yield stress values, nevertheless, the inclination was rapid. According to Laser Confocal Scanning Microscopy (LCSM), the presence of GO improved the development of flocculation structures that were comparatively looser than the primary flocculation structures of cement paste [115]. The rheological and piezoresistive properties of the blended paste of cement with different percentages of GO and nano limestone powder (NLP) were studied in a similar way [50]. The rheological parameters were investigated using four separate rheological models. A rise in the GO content was associated with a higher yield stress and plastic viscosity. On the other hand, these values were decreased as the S-P content increased. At higher shear speeds, the plastic viscosity was decreased, and the yield stress was improved [50]. According to numerous reports, the addition of GO significantly decreases the fluidity of cement pastes. However, by mixing an appropriate amount of FA with the GO, this consequence can be reduced. FA is better for improving the fluidity of GO cement pastes because of its “ball effect” and lower water demand [9]. The effect of silica fume (SF) and GO-encapsulated silica fume (GOSF) in cement paste was investigated, and similar findings of reduced fluidity due to free water entrapment were obtained due to the negatively charged GO interaction with cement. The inclusion of SF and GOSF, on the other hand, increased the fluidity marginally.

The hypothesis was supported by the synergetic effect of SF’s large surface area and the high surface activity of GO sheets (GOS). With an increase in the SF and GOSF material, the yield stress and plastic viscosity in the Bingham flow model increased significantly. The hysteresis loop was also shaped in an upward and downward curve, demonstrating the cement pastes’ thixotropic action when mixed with NGO combinations [116]. With the addition of GO, the hydration rate of the cement paste increased while the workability was decreased. The formation of calcium cation agglomerates by chemical cross-linking of GOS was used to support the hypothesis. These agglomerates entrapped the free water, reducing the matrix’s flowability [55,117]. The addition of GO to cement composites increased the compressive, tensile, and flexural strength of the materials by enhancing the microstructure [55]. In 3D printing, a mixture of GO and SF or FA may help to maintain fluidity to a certain degree [118]. Inadequate MWCNT dispersion can affect cementitious materials’ workability and limit the effectiveness of nanocomposites [119]. However, a uniform dispersion through sonication or the use of a commercially available surfactant can considerably enhance the rheological and MPs [120]. However, certain surfactants can have a negative effect on cement hydration kinetics by speeding up or slowing down chemical reactions [121]. According to some reports, the addition of 0.05%–2% CNTs to cement pastes decreased their workability and improved their viscosity. With a decrease in temperature, the total time for exothermic reactions may be extended. By NTs, the initial and final setting times were increased [122,123]. Table 3 summarizes the influence of NS on the cement paste strength reported in previous studies.

In the fresh state, the slump test measures the workability and flowability and determines the quality of flow [133]. Using 2% Nano-SiO_2_, reduced flowability of the slump by 60 mm compared with the regulated flowability of slump of 220 mm at a similar water percentage. With a 2% SP dosage, the slump flow was minimized by 90 mm compared with the reference slump flow of 220 mm [134,135]. However, due to the high water–binder ratio (w/b) of 0.45, the slump flow of 1.5% NS integrated cement pastes revealed a slump improvement of 6.5 mm over the reference slump of 72 mm [136]. The Van-der-Waals forces and the high SSA of nanoscale materials cause them to clump together. The inclusion of NSd materials in cementitious materials reduced their slump. Slump is reduced by 50% in traditional concrete with NS content varying from 2% to 4% compared with the control mix [59]. This coincides with a related point in this study regarding reduced fluidity [137]. In hybrid fiber-reinforced concrete containing bottom ash, adding NS reduced the flow behavior [138]. When a higher dose of NS and NLP was added to concrete mixes, the flowability was decreased to the same extent [134,139,140]. When 0.1% of polypropylene fibers (PPF) were substituted with 3% NS in 0.48 w/b concrete with 0.5% polycarboxylate ether (P-C-E) chemical admixtures, the workability was reduced by 15%. With the same w/b and P-C-E admixture material, the cumulative effect of 0.1% PPF and 3% NS decreased the workability by 30% compared with the 0.1% PPF mixed concrete and by 17% compared with 3% NS mixed concrete. When 3% NS was substituted with 3% NA in the same concrete with a 0.1% PPF, the workability decrease was remarkable, i.e., 33% [141]. The flow characteristics of fresh SCC mixed with NS and SF were reduced, but the quality of the fresh SCC increased significantly [142].

**Figure 4:**
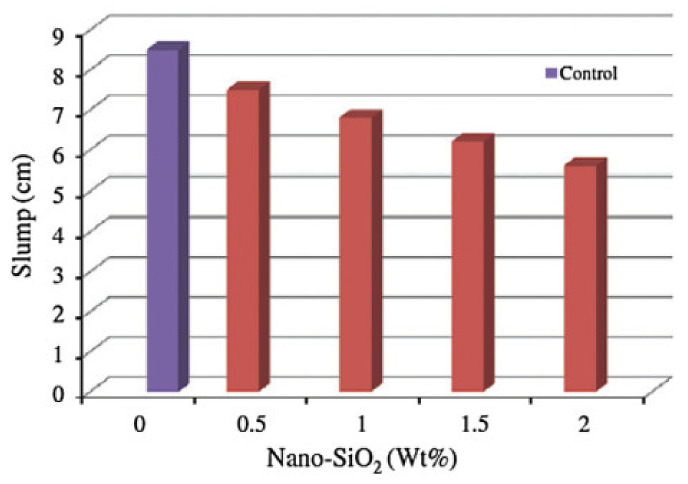
The influence of NS on the workability of concrete [86].

NS’s addition to concrete having a 15% FA resulted in a slump loss [143]. For concrete with 6% NS proposed for patch refurbishing of rigid pavements (with 0.38 w/c and having 15% to 30% FA as a cement replacement), the slump loss ranged from 55 to 80 mm, while for concrete prepared with cement that excluded NS, the slump loss ranged from 75 mm to 100 mm [144]. Similarly, at a constant SP ratio, a significant slump loss was observed in concrete mixes containing NS [145]. Incorporating 1% to 5% NA (30 wt% dispersed suspensions in water) with a normal 15 nm diameter resulted in a small improvement in the slump by 2% compared with the normal slump of 24.5 cm at 0.33 w/b and a 0.8% SP dose [146]. A low w/b of 0.16 to 0.17 in concrete mixtures with the addition of 1.0% NS by varying NLP from 1.0% to 3.0% resulted in a slump reduction by 20% and 34%, respectively. Additionally, maintaining 3.0% NLP with NS contents ranging from 0.5% to 1.5% resulted in slump flow reduction of 23 and 35%, respectively. The loss in slump is because of NMs’ large surface area, which attracts water and leaves the minimum amount of free water to contribute to the flowability of the slump [59,147]. The workability of cement mortars containing Nano-Fe_2_O_3_ at 0.5%, 1%, 1.5%, and 2% by weight of cement, at a w/b of 0.4, decreased as the nano-Fe_2_O_3_ proportion was increased [148]. However, the resulting slump in PVA-fiber-admixed nano-Fe_2_O_3_ changed by 1%, 65 mm when the fiber content was doubled, and the slump was reduced by 15 mm, a 23.07% reduction [149]. Similarly, the addition of NSd materials to cement-based materials changed the consistency properties of the cement matrix [150]. Compared with other NMs used in cementitious composites, the addition of Nano-Fe_3_O_4_ did not affect the consistency of fresh mortars due to its higher hydrophobic nature [151]. Mixtures for workability and setting time information were tested to study the new concretes. At weight levels of 0%, 0.5%, 1%, 1.5%, and 2%, NS was used to partially substitute for the cement. The results revealed that as the amount of NS in the concrete increased, the concrete’s workability decreased (refer to Figure 4). The initial and final setting times were decreased as the NS content increased [86].

The most common NPs, such as NS and nano titanium dioxide (NTD), are mainly porous and have a normal hydrophilic nature that is utilized in producing the concrete [152]. As a result, a large SSA and greater water demand minimize the amount of water available for hydration, resulting in reduced workability [153]. When 3% Fe_2_O_3_ was substituted with magnesium phosphate cement at a w/b of 0.13 and a retarder dose of 0.1%, the flowability of concrete was decreased by 35 mm compared with the reference flowability of 245 mm [138]. However, the microstructural and MPs of cement composites containing 1%, 3%, and 5% Fe_2_O_3_ (sonicated in water for 1 min to achieve uniform dispersion) did not affect the workability of fresh mortars [60,154]. On the other hand, the incorporation of NC at 2%, 3%, 5%, and 7% content by weight in cementitious grouts at a w/b of 1 demonstrated an inverse relationship with flowability and showed an improved flowability of 7.8%, 12.5%, 19.1%, and 26.5%, respectively, compared with the reference grout [155]. Similarly, upon adding micro and NSd metakaolin to ultra-high-performance concrete (UHPC), the workability was decreased by 14 mm and the 9% NSd metakaolin dosage equated to 155 mm at 1% replacement [11,156,157]. The effect of other NMs, such as NA and Nano CaCO_3_, in mixtures with fibers on the flow behavior of concretes and mortars must be investigated to produce composites for repair purposes [10].

## 4. Mechanical Properties

**Figure 5:**
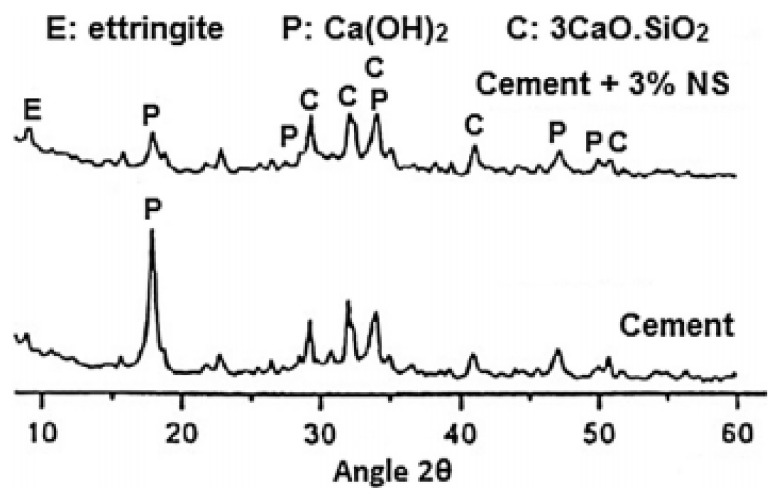
Interaction between NS and Ca(OH)_2_ at the interface between the paste and the aggregate [126].

In cement pastes, NS was used to partly substitute for cement at 0%, 1%, 2%, 3%, and 5% by weight. NS’s addition improved both the compressive and bond strength at the ages of 1, 3, 28, and 60 days. After 28 days, the compressive strength increased by 19.57%, 20.96%, 23.23%, and 24.75% at replacement levels of 1%, 2%, 3%, and 5%, respectively. Because of the pozzolanic activity of NS, which can convert the Ca(OH)_2_ produced by cement hydration to C-S-H, strengths increased as the NS content in the cement paste increased (Figure 5) [126].

### 4.1. Compressive Strength

According to several experimental studies, the use of SiO_2_ NPs in concrete increased its compressive strength to some extent. Early age compressive strength was achieved after incorporating 2% NSd silica into a high-volume FA concrete [59]. As the NSd silica particles enhanced the FA’s activation, the equal consequence of improved initial age compressive strength with a higher FA content in concrete was found [39]. A substantial increase in strength gain was observed in 7-day cured concrete samples containing 3% to 5% NSd silica content [158]. The incorporation of 5% NS enhanced the compressive strength of concrete by 30%, while NS above 5% decreased the compressive strength [141,159]. The integration of NS into FA concrete has shown improvement in strengths with age, but not in slag-powder-integrated concretes [160]. On the other hand, the NSd silica content in pozzolanic concrete should be between 0.5% and 1% [161]. The addition of NA in the range of 2% to 3% increased the compressive strength by 3% to 5% in fiber-reinforced concrete and resulted in a notable bond strength development with fibers, thereby enhancing the MPs of fiber-reinforced concrete [162,163]. From 7 days to 90 days, NA substitution by 1%, 2%, and 3% by cement weight at a w/b ratio of 0.48 resulted in an increase of 2.6% to 9% in compressive strength [164]. This pattern was explained by experimental analysis, mix preparation technology, and optimized NA mix amounts [165]. At 0.25 w/b, NM hybridization, such as 10% NS and 5% NA, in cementitious materials increased the compressive strength from 35 MPa at 3 days to 55 MPa after 28 days. Because of the reduced number of capillary pores, certain combinations of NS and NA increased the compressive strength and densified the Interfacial Transition Zone (ITZ). The ITZ’s densifying effect is not noticeable with a mono combination by just using NS [10].

Incorporation of NLP into concrete systems under standard curing conditions had a limited impact on UHPC mixtures’ strength development in the first 3 to 7 days but had a significant impact on the strength achieved from 7 to 28 days [166]. NLP incorporation by 1%–2% increased the compressive strength by 13%–18% under standard curing conditions [158]. The use of 1.6%–4.8% NLP in the concrete increased the compressive strength by 3 to 15 MPa (depending on the curing age), which was 8%–18% higher than the plain mix. This supports the research findings on UHPC with 3% NLP addition, which showed an increase in the compressive strength of 11% to 17% compared with the plain samples. NLP substituted 2.5% to 5% of the cement in the UHPC mixture, resulting in a 32% to 75% rise in 1-day strength over the control mixture [134]. Because of its high surface energy, incorporating NLP into cementitious materials will noticeably increase the heat of hydration. It also strengthens the early age MPs of the UHPC matrix by increasing the medium’s filling density and serving as a nucleation site for C-S-H gel formation [10].

**Figure 6:**
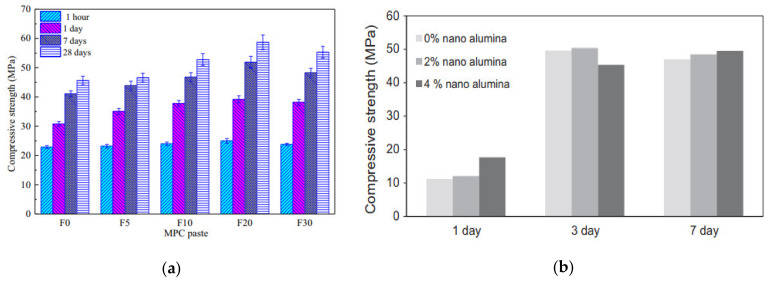
Compressive strength: (**a**) MPC paste varies with different dosages of Fe_2_O_3_ powder [28]; (**b**) Cement replaced with 0%, 2%, and 4% of Nano-Al_2_O_3_ [167].

Figure 6a depicts the outcomes of the compressive strength tests. After one hour, the compressive strength of the reference sample (F0) was 22.9 MPa, and it was improved to 30.8 MPa, 41.1 MPa, and 45.6 MPa after 1 day, 7 days, and 28 days, respectively. The one-hour-hardened specimen’s compressive strength increased from 22.9 MPa (F0) to 25.0 MPa (F20) with consecutive additions of Fe_2_O_3_ powder to the MPC paste but decreased to 23.8 MPa (F30) with further addition. F20, with a 20% Fe_2_O_3_ powder, had the highest intensity across the entire curing cycle of the five classes. Its compressive strength was 39.2 MPa, 51.9 MPa, and 58.7 MPa after 1 day, 7 days, and 28 days of curing, respectively. The findings revealed that adding a suitable quantity of Fe_2_O_3_ powder to the MPC system significantly increased the compressive strength [28]. A cement-based paste’s compressive strength with the addition of 2% and 4% of Nano-Al_2_O_3_ improved significantly at 1 day and 7 days. The compressive strength difference was minimal at 1, 3, and 7 days, and the addition of Nano-Al_2_O_3_ to the cement did not affect it. However, a cement paste with nano-Al_2_O_3_ may have a more substantial long-term strength. It is, therefore, necessary to investigate the compressive strength shown in Figure 6b [167]. The addition of nano-Fe_2_O_3_ at 3%, 5%, and 10% to a cementitious composite improved the compressive strength. With 10% Nano-Fe_2_O_3_ material in cement mortar, an increase of 66.81%, 69.76%, and 25.20% in the compressive strength was found at 7, 14, and 28 days, respectively, relative to the normal mix. Similar increases in compressive strength were observed as Nano-Fe_2_O_3_ was used in 0.5% to 5% ratios in mixes of concrete and mortar [148,168]. After 90 days, using nano-ferrite at an optimal proportion of 2% resulted in the improvement of the compressive strength by 17% over the control blend. Incorporating NA into the cement matrix has led to a remarkably denser microstructure owing to the improved compactness of the ITZ and the reduced porosity [12].

**Figure 7:**
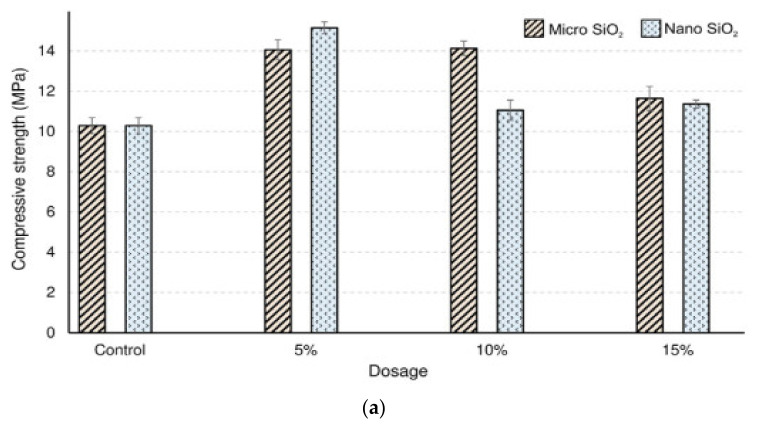

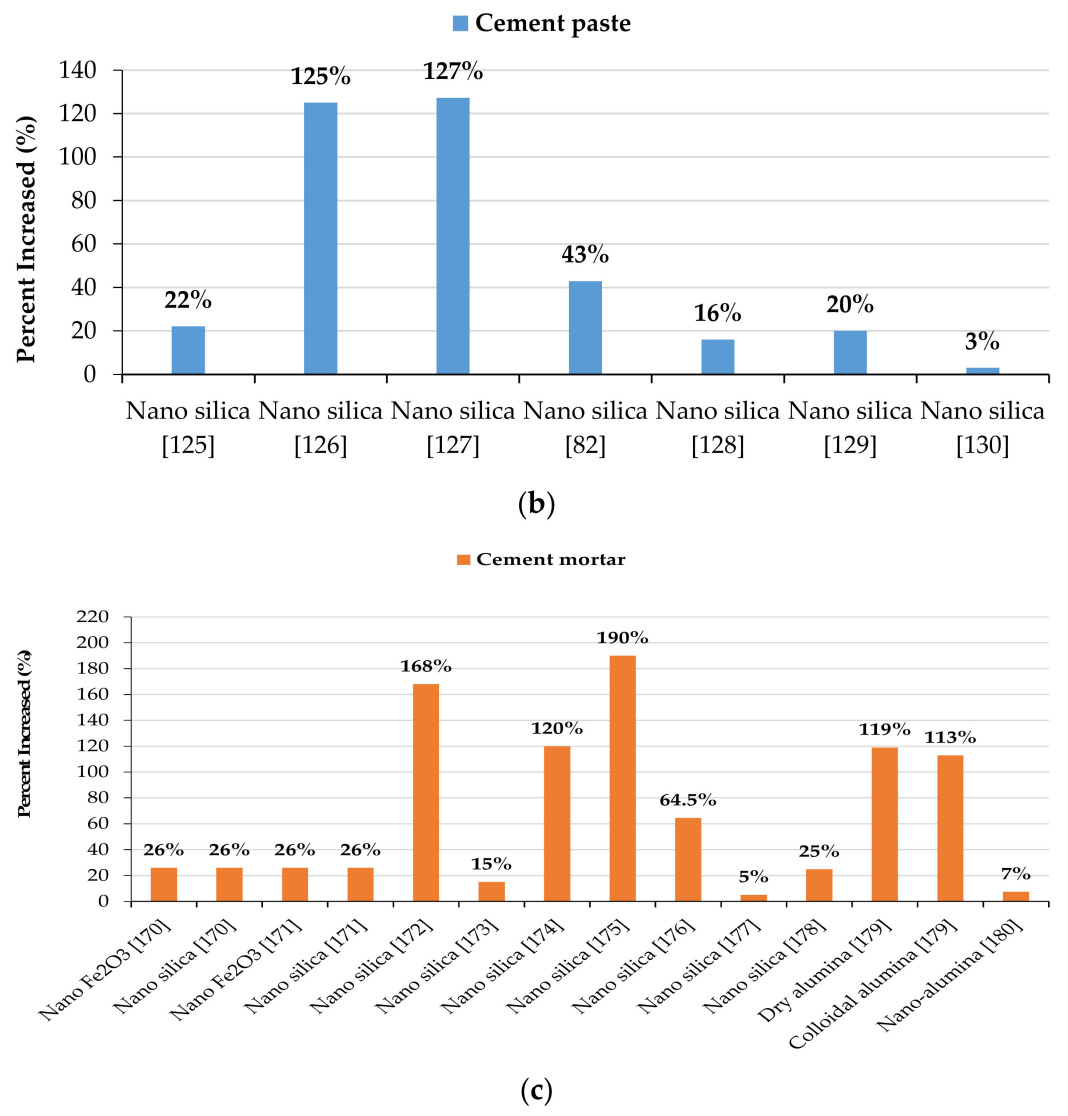
(**a**) Compressive strength in the presence of micro and nano silica particles [169]; (**b**) Compressive strength percentage increment for cement paste [82,125,126,127,128,129,130]; (**c**) Compressive strength percentage increment for cement mortar [170,171,172,173,174,175,176,177,178,179,180].

The results from the compressive strength tests are presented in Figure 7. Generally, the compressive strength was higher for mortar samples with micro silica and NS, regardless of the dosage, compared with the compressive strength of the control samples. The highest compressive strength was observed when 5% of NS or micro silica was included in the mixture. However, with the increasing content from 5% to 15% NS, the compressive strength decreased from 15.2 MPa to 11.4 MPa, while the addition of 5% to 15% micro silica decreased the compressive strength from 14.1 MPa to 11.6 MPa. This suggests that NS and micro silica can improve the compressive strength when used at lower proportions and subsequently decrease the compressive strength when increasing their amounts. Overall, the addition of NS appeared to improve the compressive strength better than the micro silica, where a relatively higher compressive strength of 15.2 MPa was achieved with 5% NS [169].

**Figure 8:**
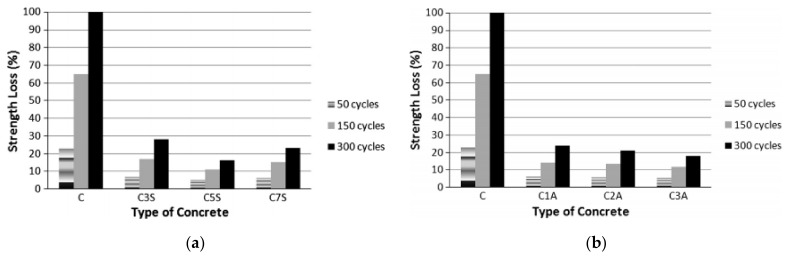
Strength loss of samples having: (**a**) NS; (**b**) NA [141].

The effect of NS and NA on concrete’s compressive strength is illustrated in Figure 8. As seen in Figure 8a,b, the strength loss of NP-containing concrete is significantly less than the control concrete Figure 8b. Concrete comprising 3% NS (by weight of cementitious material) reported just a 28% strength loss after 300 cycles of freezing and thawing, while the control concrete showed a 100% strength loss. The compressive strength of concrete comprising NS decreased from 28% to 16.28% as the NS content increased from 3% to 5% after 300 cycles of freezing and thawing, then increased from 16.28% to 23.20% as the NS content increased from 5% to 7% after 300 cycles of freezing and thawing. The percentage of strength loss in NA-containing concrete was found to be much lower than that in the control concrete, dropping from 23.84% to 18.19% as the NA content increased from 1% to 3%. [141].

**Figure 9:**
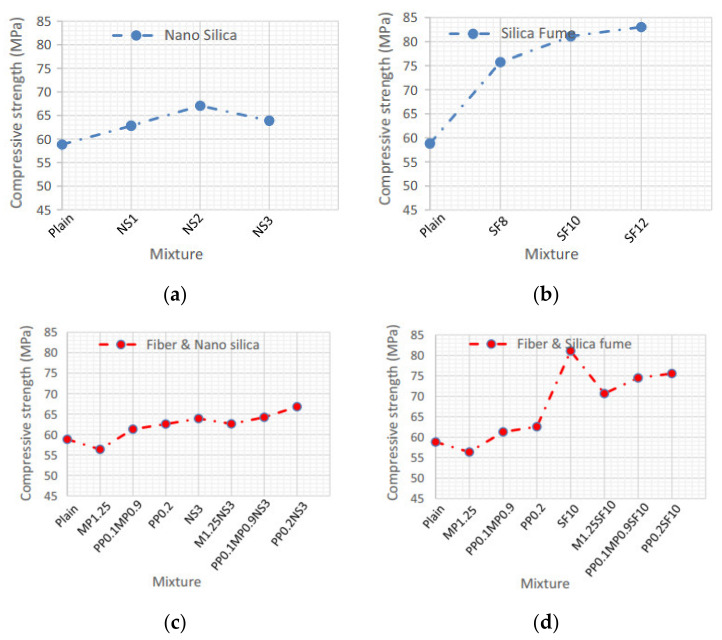
Compressive strength of concrete having: (**a**) NS; (**b**) SF; (**c**) fibers and NS; and (**d**) fibers and SF [181].

Figure 9 illustrates the compression test findings of concrete specimens. Compared with the plain concrete specimens, the sample comprising 1%, 2%, and 3% NS had an increase in compressive strength of 6.8%, 14%, and 8.6%, respectively. In comparison, samples having 8%, 10%, and 12% SF had an increase in compressive strength of 28.7%, 37.9%, and 41.1%, respectively. The compressive strength of plain concrete and concretes comprising the optimal percentage of NS and fibers was improved when the optimal percentage of PPF was coupled with that of NS (PP0.2NS3) and when the optimal percentage of hybrid fibers was combined with that of NS (PP0.1MP0.9NS3) [181].

### 4.2. Flexural Strength

The addition of NMs to cementitious materials resulted in improved flexural strength. NS as a substitute for up to 3% of the cement enhanced the specimens’ flexural strength, while a 4% replacement level reduced the flexural strength [12]. The flexural strength outcome in binary blended concrete with 1% NS as a cement replacement showed a noticeable contrast [87]. After adding a hybridization of NPs, including NS and NLP, to an UHPC mixture at 7 days and 28 days, with a content limit of 1%, the small quantity of NS and NLP increased the flexural strength, which started to decrease as the quantity of NPs increased [142]. On the other hand, the concrete’s flexural strength with a 3% NS substitution was weaker than that of the control concrete [182]. At the 1% cement replacement level, the noticed flexural strength increased at 7 to 90 days, from 5.3 MPa to 6 MPa, respectively. However, at the 2.0% cement replacement level, the noted flexural strength decreased from 17% to 20% over the same period. However, replacing nano Fe_2_O_3_ with 1% cement resulted in a lower flexural strength enhancement [168,183]. While the addition of nano ferrite at 2% in cementitious materials increased the flexural strength more than with the higher percentage addition, the higher percentage incorporations resulted in a decrease in flexural strength [12]. As NS and nano ferrite were added to the high-strength concrete (HSC), they increased its flexural strength by 23% over the control concrete [133]. Furthermore, by combining NS and steel fibers, the flexural strength of hybrid fiber-reinforced H-P-C was significantly improved [184]. MPC’s flexural strength and fracture energy were improved by adding PVA fibers [28,185].

**Figure 10:**
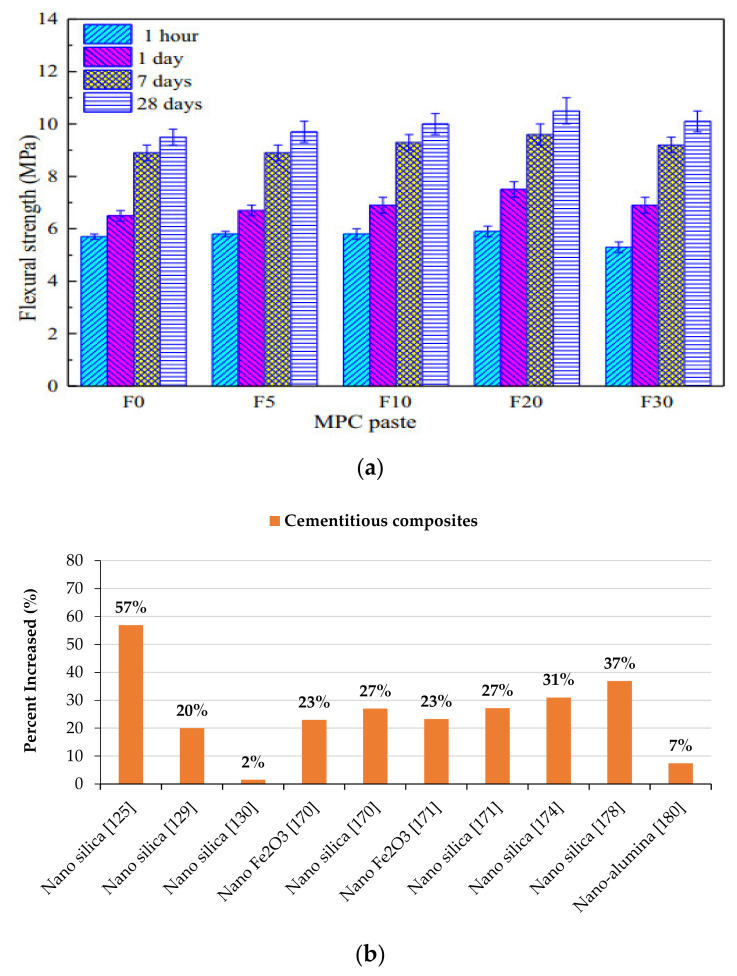
(**a**) MPC paste’s flexural strength with different dosages of Fe_2_O_3_ powder [28]; (**b**) Flexure strength percentage increment for cementitious composites [125,129,130,170,171,174,178,180].

The influence of Fe_2_O_3_ powder’s inclusion on the cement paste’s flexural strength is identical to its effect on compressive strength, as seen in Figure 10. When the additive amount was 20% Fe_2_O_3_ powder, the hardened sample’s flexural strength at 28 days was improved from 9.5 MPa (F0) to 10.5 MPa (F20). However, strength was decreased to 10.1 MPa when the additive level was 30% Fe_2_O_3_ powder. After curing for 1 h, 1 day, 7 days, and 28 days, the researchers discovered that the inclusion of 20% Fe_2_O_3_ powder resulted in flexural strengths of 5.9 MPa, 7.5 MPa, 9.6 MPa, and 10.5 MPa, respectively. The flexural strength decreased slightly with further addition of Fe_2_O_3_ powder to a maximum of 30% [28].

Similarly, the incorporation of glass fibers into concrete at 2.5% of the optimum dose affected the flexural strength development similarly to the compressive strength [186]. Furthermore, a 0.5% basalt fiber (BF) addition to MPC resulted in a compressive strength of 49.42 MPa and flexural strength of 5.31 MPa after 28 days [134]. Finer fibers improve the flexural strength and fractured energy more than thicker fibers [185]. The rapid consumption of the hydration product Ca(OH)_2_ improved the development of the flexural strength of cementitious composites containing NMs. One aspect of the restrictive fiber effectiveness in cementitious materials is the inability to form a strong bond between fibers and the cement matrix [163]. The addition of NMs to the cementitious materials affects their flexural strength. Nano CaCO_3_ influenced the cementitious material with a significantly greater improvement in flexural strength than NS incorporation at a similar dosage of 1%. The nano meta clay NS in its colloidal form at a smaller amount more effectively enhanced the cement matrix’s flexural strength than the powder type [10].

### 4.3. Tensile Strength

**Figure 11:**
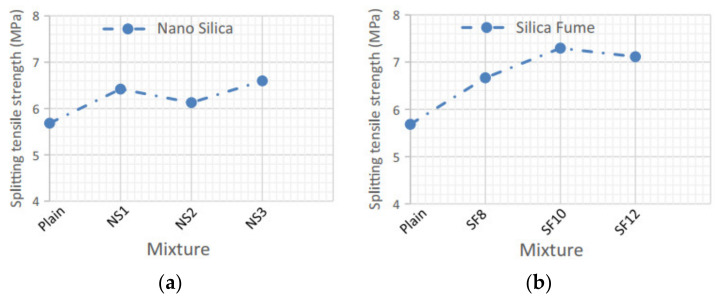

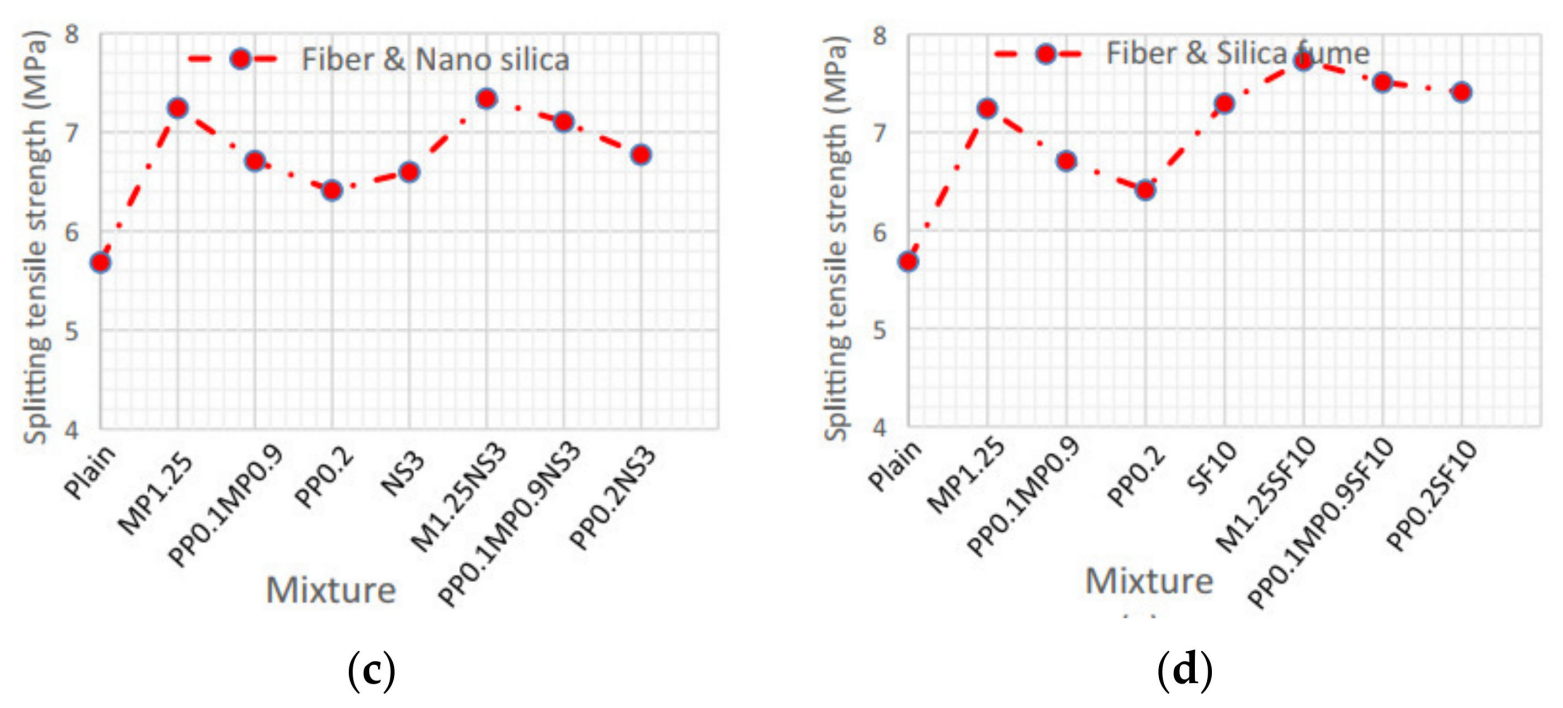
Splitting tensile strength for concrete containing: (**a**) NS; (**b**) SF; (**c**) fibers and NS; and (**d**) fibers and SF [181].

NMs have outstanding MPs due to their NPs’ volume, surface, and quantum effects. When NPs were used to refine the grain of a common substance, they provide an intergranular or intragranular arrangement that strengthens the grain boundary and the material’s MPs [187,188,189]. Compressive strength, bending ability, and breaking tensile strength can also be enhanced by using 3% (by wt) CaCO_3_ in concrete [190]. Adding a 3% nano oil palm empty fruit string filler to kenaf epoxy composites increased their tensile efficiency and elongation at the split and affected their strength considerably [191]. Nanocomposites with increased tensile strength, flexural strength, toughness, and specific wear rate can be made by adding 2% (by wt) NC to an epoxy resin matrix [192]. When the amount of GO in the cement matrix was 0.03% (by wt), the flexural strength, tensile strength, and compressive strength increased noticeably [193]. The material’s compressive and flexural strengths were enhanced by 33% and 41%, respectively, when the GO content reached 0.05% [194]. Different wet states may also affect the MPs of NMs. The tensile strength and total load capacity of a PVA/chitosan/NZ hydrogel were improved when the NZ content increased under dry conditions, while the elongation at failure first grew and then dropped. In the wet state, the tensile strength and total load capability declined markedly, while the elongation at failure was enhanced [195]. The elongation at failure rose markedly as the NMs were sonicated for longer than 30 min, but the tensile strength was reduced. When the treatment period was increased to one hour, further pores appeared on the material’s surface, reducing the material’s MPs [196]. Figure 11 illustrates tensile strength results on specimens containing various fibers, NS, SF, and a fiber–pozzolanic mixture. Since HSC includes a higher proportion of SF, the SF particles have a greater effect on the split tensile strength than NS particles, as seen in Figure 11a,b. Furthermore, an unfinished hydration reaction exists due to the low w/c of HSC and the higher absorption of water by NS because of the high SSA, resulting in a specimen with NS particles providing a lower effect on strength than a specimen with SF particles [181].

## 5. Durability Performance

### 5.1. Shrinkage

Every cementitious product shrinks because of water loss from the materials, resulting in a reduction in volume. At 90 days, RHA-incorporated mortars showed dry shrinkage ranges from 801 to 843 micro strains, which was minimized by 5% to 12% when the RHA incorporation was increased from 10% to 30%. There was also less shrinkage when compared with the control sample [197]. When used together or separately, RHA and PPF in mortars significantly decrease drying shrinkage. PPF and RHA seem to work together to minimize mortars’ drying shrinkage [138]. Previous research on high-volume FA concrete reinforced with PPF found that it had the same low drying shrinkage properties [198]. Similarly, as the volume fraction of fibers in PPF composites increased, dry shrinkage reduced [199,200]. PPF used to monitor actions at the micro-level by connecting and finishing fineness cracks backed up this conclusion [160]. In contrast to the control sample, NS and SF caused substantial shrinkage, where samples containing NS showed higher shrinkage due to chemical shrinkage [201]. Another related study found that, by replacing 1.75%, 3.5%, and 7% cement with NS, shrinkage was increased by 80% at 7 days and reduced by 54% at 28 days due to early acceleration [183]. Compared with a control mix prepared with super-absorbent polymers (SAPs) alone, the use of SAPs combined with an SP in the cementitious material resulted in a 75% reduction in shrinkage [39]. Due to an early age reaction with pozzolanic material, the total shrinkage was observed to improve as the NS content increased [202]. Compared with the control group, the reduction in shrinkage was marginal when up to 3% of the cement was replaced with NLP [203]. When the NLP content was increased to 3%, the autogenous shrinkage decreased due to the agglomeration on the higher surface area of NPs [204]. Similar expansion patterns were observed and related to the SAP ratio and an advanced absorption capability [173].

In contrast to the normal sample, the addition of nano synthetic fibers to a mortar sample at a 0.26% volume ratio decreased the cracked area of plastic shrinkage by 36%. Compared with a mix of the same amount of steel fibers, a mortar with an NF admixture had a 33.6% reduction in crack area [205]. As equated to NS with a particle size of 40 nm, NS with a particle size of 12 to 20 nm showed higher shrinkage values. However, cement mortar with 40 nm NS had the least dry shrinkage owing to a higher packing density and a smaller NS particle size [83]. This is consistent with findings that show that high porosity significantly impacts the shrinkage and water absorption of cement mortar [206]. The particle size of NS influences the essential properties of cementitious materials, such as resistance to abrasion, permeability, dry shrinkage, bond strength, and cracks. The properties of cementitious materials can be improved by using NS particles with a size of 40 nm [83].

### 5.2. Pores

As NMs are integrated into a cementitious composite, the composite’s porosity (or number of permeable voids) is reduced [16]. Nano magnetite’s introduction into mortar mixtures resulted in a porosity reduction of up to 6% [155]. The filler effect of nano magnetite combined with the accelerated formation of hydration products contributes to the microstructure’s densification, resulting in a reduction in the number of permeable voids. The effect of different nano magnetite percentages on mortar porosity is pronounced. When NS was used as a 7% substitute of cement in cementitious composites, the porosity of the composites was reduced [180]. This finding is consistent with those of a study that found reduced porosity by 27% by adding 2% NS to the mortar [207]. Similarly, using NC in hemp-fiber-reinforced composites significantly decreased the porosity. The formation of more hydration products resulted in the densification of the microstructure, which was linked to the composites’ porosity. However, 1% NC was determined to be the optimal dosage for lower porosity, as a dosage greater than 1% increased the porosity [208]. To minimize a mortar’s porosity, an optimal dose of 1% NS is used [172]. NTI can be used in fiber-reinforced cementitious composites to minimize the pore content. Concerning the effect of nano magnetite on porosity, it was found that the optimal dosage of NTI to decrease the porosity of cementitious composite is 1%. This means that before NMs are used on a broad scale, experimental testing to assess the appropriate dose must be performed [209].

**Figure 12:**
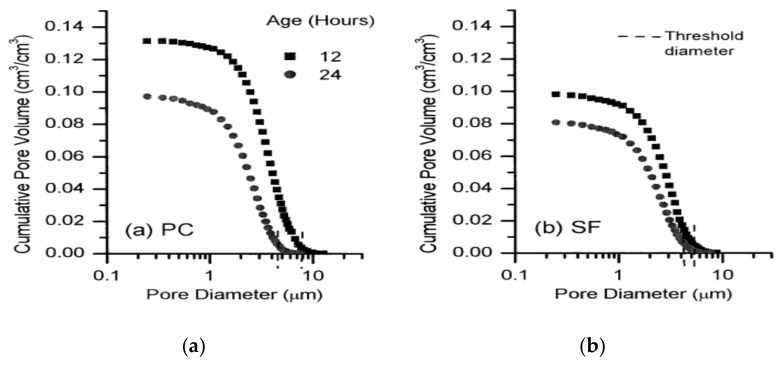
Coarse capillary pore size distributions at early ages: (**a**) Portland cement concrete; (**b**) SF concrete [210].

Figure 12 shows coarse capillary pore size divisions in HSC with and without SF within the cement paste phase. SF-containing composites had smaller pores than the plain composites (without SF) at 12 and 24 h. In SF-containing composites, the diameter from which porosity starts to rise abruptly as the diameter of the pore declines at 12 h is smaller than in normal concretes. SF’s narrower threshold diameter indicated that the binder grains in the composite were packed more densely. The primary porosity gap between the plain composite and the 10% SF-containing composite was very minimal by thickness. It can be concluded by utilizing SF (density: 2.2 g/cm^3^) and cement (density: 3.15 g/cm^3^) in composites, the difference between the porosity measured by mercury intrusion porosimetry (MIP) and that measured by picture analysis (Figure 12) showed the sizes of finer pores to be less than 0.2 µm if there was just a small variation in the overall porosity between composites with and without SF at early ages. The results in Figure 12 suggest that SF-containing composites might have finer pores than composites without SF. The overall porosity could be higher than the porosity of the larger pores counted in the image of the sample [210].

### 5.3. Porosity

MIP is extensively used to determine the pore structure in porous materials and to study the existence of interlinked pores because of its ease of use, quickness, and capacity to measure a broad range of pore diameters [210]. Increasing the content of NS resulted in a 4% enhancement in micropores and a 40% decrease in essential pores after incorporating 6% colloidal NS, successfully triggering pozzolanic reactions and inducing a filler impact [182]. The pore structure determines the permeability properties of cementitious composites. The characteristics show how easily different harmful materials can penetrate the composite. In general, the addition of NMs decreased the permeability of the corresponding composite because of the pore refinement induced by the NMs’ filler ability as well as its pozzolanic reaction [86,209,211]. This study’s findings are in line with the literature [212]. There was no significant effect of NS in its slurry form on the mixture’s porosity compared with the reference specimen’s porosity of 13.5%. However, it resulted in a 15% decrease in the number of capillary pores (>50 nm) and a 32% increase in the number of medium capillary pores (10–50 nm). This could be due to NS’s ability to refine pores and fill them [201]. NS of 10 to 20 nm in size is efficient at pore filling and accelerating pozzolanic reactions, resulting in improved cement mortar properties such as a dense microstructure, increased compressive strength, and decreased permeability [213,214]. Furthermore, SEM analysis revealed a more refined microstructure than the reference mortar [82]. It was discovered that adding NMs to cement slurry enhanced the pore structure and density of hardened cement paste (Table 4). Compared with plain cement, the average pore size and total porosity of nano cement were reduced by 16.9% and 25.5%, respectively [215].

## 6. 3D Printing of Cementitious Composites

Cementitious composites used for 3D printing can be considered a filament/ink material that must satisfy certain criteria to be extruded using a pipe pump–nozzle device. In the literature, three separate extrusion-based printing regimes have been suggested. One is extrusion in which the cross-sectional dimensions of the filament and nozzle are the same. This technique is commonly used to print ultra-stiff materials. The extrusion of flowable materials without external forces is known as free-flow extrusion. The material flows naturally from the nozzle using just gravity energy. It is worth noting that the material flows before the shear yield stress of printable materials, and the stress caused by gravity achieves equilibrium. Non-equal filament/nozzle cross-section dimensions, coupled with external feedback forces, including friction, may be used to print and deposit rigid products [216]. Filaments/nozzles with lower dimensions can produce material shear staggering, i.e., the nozzle dimensions between the print head and the filament result in different localizations of the materials, which induce material shifts throughout printing [217,218].

Additionally, at maximum consistency, a large amount of plastic hydration/shrinkage was found in cementitious composites up to the stage where it becomes solid. The mixture of composites’ properties should be expanded until successive levels of composite are applied to create a durable mix, which is particularly important because of fresh concrete’s vulnerability to various environmental conditions. These include the 3D-printable characteristics of pumpability, extrudability, workability, and buildability [219]. Pumpability is characterized as a mixture’s ability to be mobilized under pressure while preserving its original properties [220]. The typical slump test and the slump flow test are commonly used to determine the pumpability index [221]. Pumpability has been measured using a tribometer, a sliding pipe rheometer, and a viscometer in other tests [222,223]. To fully comprehend the phenomenon of pumpability, these specific experimental findings must be combined. The composites’ capacity to be continuously printed through the nozzle has been tested using a ram extruder, visual observation, a 4.45 KN servo-controlled MTS system, and other uncontrolled techniques [224,225,226,227]. While visual observation can be a good starting point, it involves a high risk of human error. These studies have not proven to be the most accurate and effective alternatives for testing extrudability in 3D-printed concrete (3DPC). Open time is an important factor to consider when assessing a mixture’s workability in 3D printing. The open time is the amount of time the mix is dispensed without stopping or clogging the nozzle [228]. A rotational rheometer, the Vicat process, and a flow table were used to test the 3DPC workability [229]. When designing a 3D printing mixture, various final (hardened) properties such as bulk density, layer adhesion, MPs, durability, and shrinkage should be considered [230]. The majority of these variables are heavily influenced by the composition of the mixture and the printing process. Moisture loss can occur because 3D-printable mixtures involve high fine particle content in the formulation. The hydration process takes place in an open atmosphere due to the lack of formwork, which may cause the structure to shrink and crack [231,232]. A list of recent studies on the integration of NMs into 3D-printable composites is presented in Table 5. Non-optimal curing conditions can cause an increase in cracking in such mixtures, enhancing the penetration of water and aggressive chemicals, degrading the cement paste, and minimizing the structure’s lifetime [233]. Pore volume and distribution are essential microstructural characteristics used to understand the behavior of 3DPC, particularly in the interlayer bond, which has been described as one of the weakest sections in printed elements [230,234]. Several flat and elongated pores exist in 3DPC, especially in the inter-layer transition region [235]. In the same way, improved porosity in the interlayer zones of 3D-printed specimens, though pore volume, was not directly associated with the specimen’s tensile strength. [236]. Different amounts of fine aggregates and chemical admixtures are necessary depending upon the required strength category of the concrete, such as conventional concrete, UHPC, or 3D-printed concrete. Three cement-based mixtures and one multi-binder geopolymer were chosen to be printed [234,237,238,239]. Clay NM is another kind of material that has shown great potential in 3D printing mix modifications. Clay NPs, like NS, are considered to be effective thickening agents, which may be due to the clay particle flocculation, high water adsorption, or potential contact between ettringite and clays [240]. NC is normally used in 3DPC to modify the rheological properties and structural build-up. Purified magnesium alumino-silicate, metakaolin, kaolinite, and illite, among others, are among the clays used, each with its own set of properties [43,241].

There have been many advances in the field of NMs and nanocomposite research due to the existence of tunable properties of NMs and their composites. The field of AM has recently attracted a lot of attention because it offers a lot of potential for producing complex and extremely small designs. AM is most likely to transform future industries, ushering in the third industrial revolution. It is possible to make the latest technology accessible and more sustainable by easing the material processing requirements with the approach of using NMs in AM. There have been several advancements in the incorporation of NMs in AM for different applications in the last few years [245]. Despite cost issues, there are several hurdles to overcome before AM technology can be widely adopted. The lack of existing standards related to AM technology will effectively prohibit the use of these techniques for massive construction in the near future, particularly in European countries where strict standards are applied to the construction industry in terms of procedures and material selection. Furthermore, it has yet to be determined if AM technology provides a cost-effective fabrication process in the construction industry; thus, it is critical to assess the life-cycle costing of AM technology based on material feedstocks and printing systems [246,247]. Therefore, a cost analysis is suggested for future study.

## 7. Microstructural Properties of Nanomaterials in Composites

**Figure 13:**
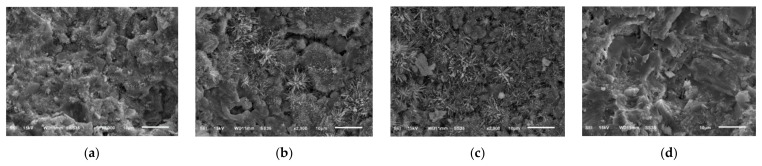
SEM images of cement paste incorporating NMs: (**a**) C-P; (**b**) CPNM2; (**c**) CPCNTs03; (**d**) CPNC2 [205].

The cement paste’s microstructure is shown in Figure 13a, and the morphology of cement-based materials with NMs, CNTs, and NC is shown in Figure 13b–d. As shown in Figure 13a, the hydration products are mostly C-S-H gels that are cross-linked into the paste. C-S-H gel and Aft are the main hydration products with NMs’ addition, as shown in Figure 13b. They are widely distributed, and the C-S-H takes the shape of a flocculent gel. The CNTs are wound and twisted together into a continuous network structure and tightly pack the hydration products, as shown in Figure 13c [248]. The C-S-H gel, Ca(OH)_2_ crystals, and other hydration products form a relatively compact microstructure, as shown in Figure 13d. The addition of NMs to cement-based materials enhanced the microstructure and compaction of the matrix compared with a C-P group [205].

**Figure 14:**
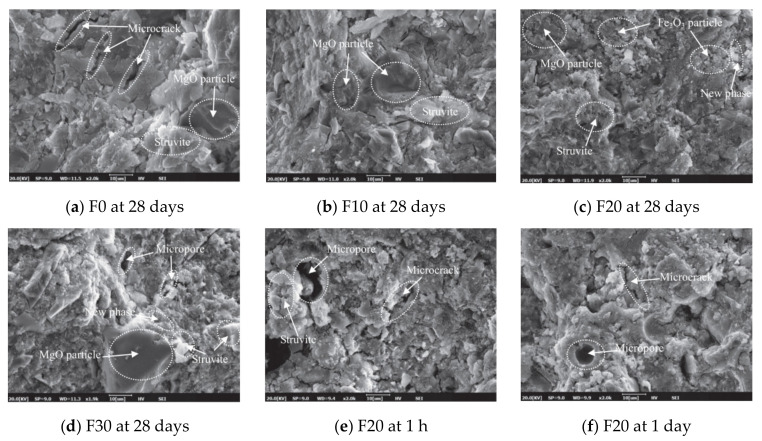
SEM images of MPC pastes with several dosages of Fe_2_O_3_ powder [28].

**Figure 15:**
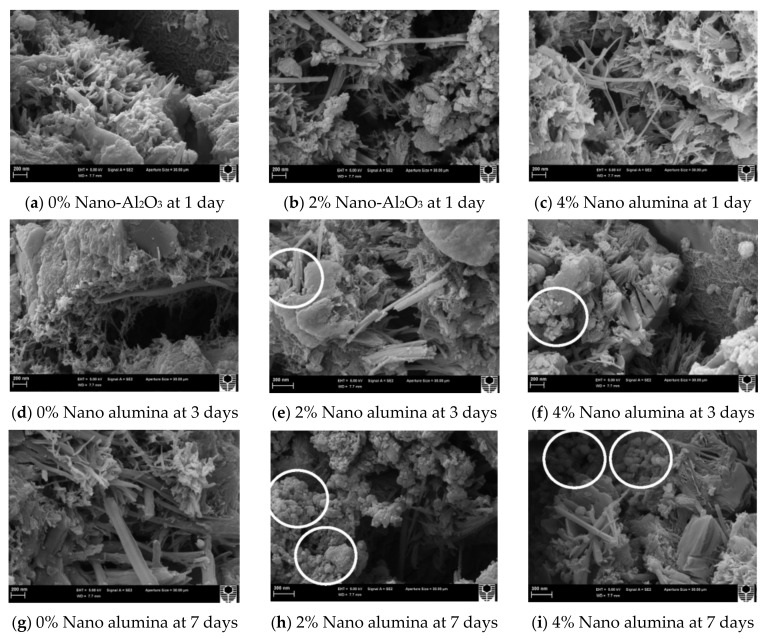
SEM images of 0%, 2%, and 4% nano-Al_2_O_3_ content mixture [167].

F20 has a more compact microstructure with fewer cracks than the other groups, as shown in Figure 14a–d. A denser microstructure typically implies that the hardened sample is stronger, and this section will support the strength test results. Due to the smaller particle size, the fluidity decreased considerably after the addition of Fe_2_O_3_ powder. A reduction of fluidity in the fresh slurry can result in the formation of a heterogeneous microstructure, as seen in F30. Since the strength is primarily due to MgO and ADP’s reaction, an excessive dose of Fe_2_O_3_ powder can reduce the binding force within the particles [28]. Figure 15a–i show SEM images of a 0%, 2%, and 4% nano-Al_2_O_3_ material mix that was cured for 1, 3, and 7 days. For the 1-day-cured samples with the 0%, 2%, and 4% nano-Al_2_O_3_ content mix, C-S-H and ettringite formation were observed. The 4% nano-Al_2_O_3_ content specimens had fewer voids than the 0% and 2% nano-Al_2_O_3_ content samples. In comparing the 0% nano-Al_2_O_3_ content specimens with the 2% and 4% nano-Al_2_O_3_ content specimens, larger portlandite crystals were found after 7 days. With the 2% and 4% nano-Al_2_O_3_ material samples, agglomerations of nano-Al_2_O_3_ particles were also observed, shown by white circles in the SEM pictures. With the inclusion of nano-Al_2_O_3_, SEM analysis shows the creation of a much denser microstructure [167].

**Figure 16:**
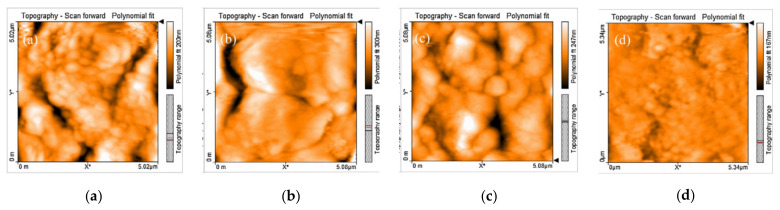
AFM analysis of concrete with: (**a**) plain cement; (**b**) nano-silica; (**c**) silica fume; (**d**) polypropylene fibers and nano-silica [181].

The plain SF and NS-containing concrete and the fiber-reinforced SF and NS-containing concrete are shown in Figure 16. Atomic force microscopy (AFM) is a micrometer-scale imaging method for observing the porosity on the concrete’s surface. As a result of their broad apparent dimensions, AFM photographs of macro-polymeric fibers cannot be obtained for examination purposes. The black color indicates the specimens’ pores in the figure. Because of the higher specific surface area and finer pozzolans, the samples with SF and NS have a lower porosity than the plain concrete sample, as shown in Figure 16a–c. In general, pozzolanic material as a cement substitute in concrete produces C-S-H gel, which decreases the porosity of the concrete through a pozzolanic reaction with the Ca(OH)_2_ produced during cement hydration. Figure 16b shows the porosity of the concrete with the optimal percentage of NS (3%), and Figure 16c shows the porosity of the concrete with the optimal ratio of SF (10%). The porosity of the specimens containing NS and SF decreases when PPF is added, as shown in Figure 16d, since the fiber surfaces are covered with a densely hydrated cement matrix. The surface of the sample with PPF and SF is approximately smooth, and its porosity is negligible, which may be due to the strong bond between the fibers and the matrix of the concrete with a high volume of SF [181].

**Figure 17:**
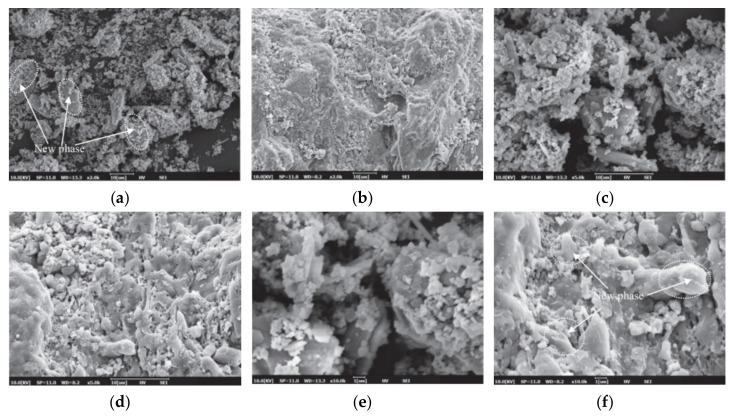
SEM images: (**a**,**c**,**e**) for NF-30; (**b**,**d**,**f**) for NF-90 [28].

**Figure 18:**
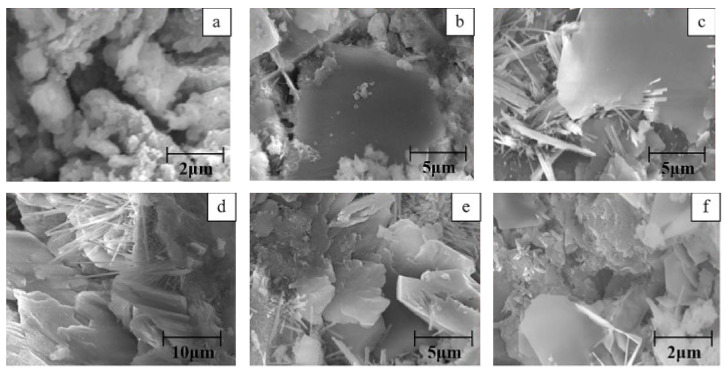
SEM of cement composites modified by different contents of GONPs: (**a**) no GONPs; (**b**) 0.01% GONPs; (**c**) 0.02% GONPs; (**d**) 0.03% GONPs; (**e**) 0.04% GONPs; (**f**) 0.05% GONPs [249].

**Figure 19:**
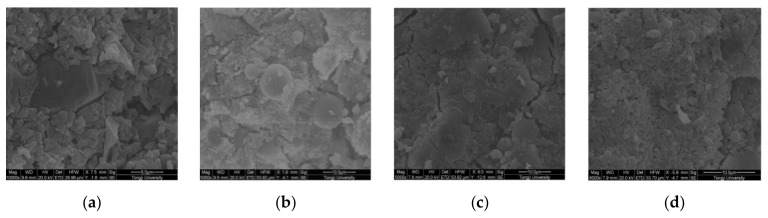
E-SEM photos of MPC samples at 28 days of curing: (**a**) for the plain sample, (**b**) for the sample with 20% FA, (**c**) for the sample with 5% SF, and (**d**) for the sample with 20% ultra-fine FA [27].

Figure 17 shows various FN90 and FN30 micrographs (2000, 5000, and 10,000). Unreacted ADP crystals with a large square shape and leftover Fe_2_O_3_ particles with a slight columnar or wedge shape were clearly observed in the FN30 images. Similar to the SEM observation of two ingredients, the microstructure of FN30, which had a weak gelling impact, showed wide gaps among the particles. In FN90, small Fe_2_O_3_ particles were present on the large square-shaped ADP crystal, and some were also embedded in it. The microstructure was extremely compact, and some new tiny column-shaped gels were discovered (marked in the images). The gaps between the residual materials were filled in by these new gels, showing a strong pore-filling effect. These cementitious gels were assumed to have a better binding strength and could closely connect the particles in this system [28]. The distribution of the hydrated phase is non-uniform, as shown in Figure 18a. There are several macropores and microcracks. The addition of graphene oxide nanoplatelets (GONPs) provides bridging between cement hydrates, modifies the microstructure, and results in an additional uniform and compact microstructure (Figure 18a–f) [249]. Figure 19 shows environmental scanning electron microscopy (E-SEM) images of MPC specimens cured for 28 days with 20% FA, 5% SF, and 20% ultrafine FA. Each specimen contains some MKP products with well-crystallized crystals, as shown in Figure 19. Comparing the plain sample (Figure 19a) with the 20% FA sample (Figure 19b) and with the 5% SF sample (Figure 19c), there are multiple microcracks. The formation of potassium chloride crystals may cause the microstructure to crack due to the expansion stress. When 20% ultra-fine FA is used in the MPC mortar, as shown in Figure 19d, the MPC specimen has fewer microcracks, and the microstructure’s compactness has improved. This finding suggests that MPC specimens with ultra-fine FA have higher strength than MPC specimens with FA or SF [27].

**Figure 20:**
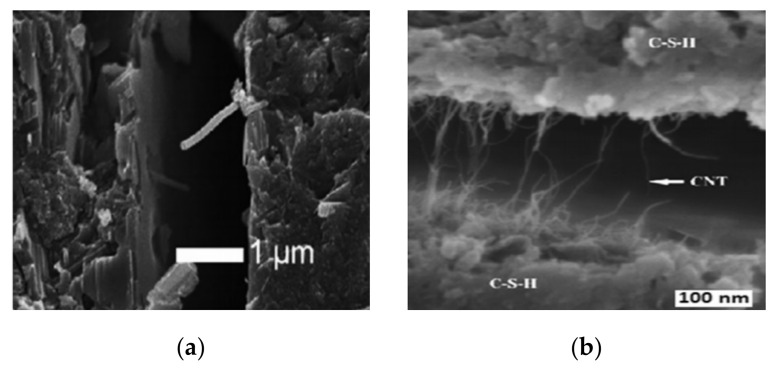
(**a**) C-NFs pulled out from a microcrack, and (**b**) bridging a crack by embedding CNTs in a cement-based composite [250].

**Figure 21:**
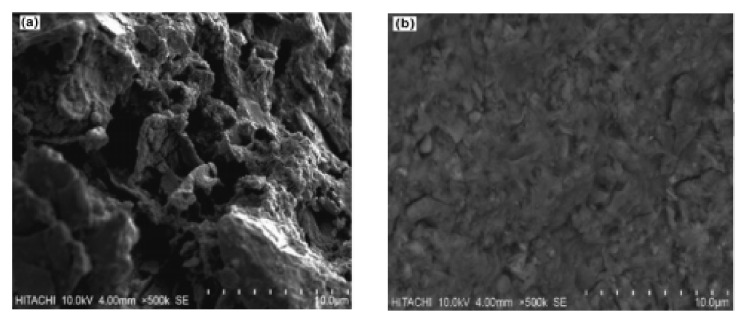
SEM images of (**a**) plain concrete and (**b**) concrete modified with 1% NS [251].

**Figure 22:**
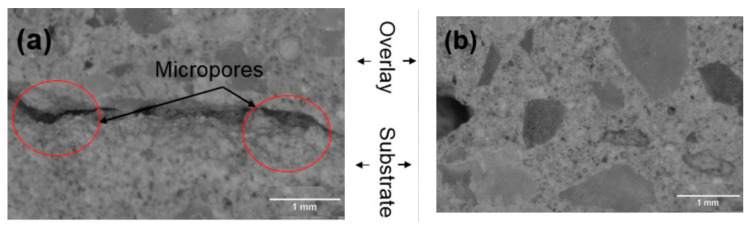
SEM images: (**a**) specimens with 0.5 wt% NC and (**b**) a control specimen [253].

CNTs, carbon nanofibers (CNFs), and nano graphite platelets (NGPs) are the three most common forms of carbon fibers used in multifunctional cementitious composites. CNTs have a hollow cylindrical nanostructure and are classified as SWCNTs or MWCNTs, depending on how many rolled graphene layers they contain. CNFs are quasi-one-dimensional carbon materials with a diameter somewhere between that of CNTs and carbon fibers, whereas NGPs are graphite-based conducting materials. GO is a single layer of NGPs that have been oxidized. Figure 20 depicts CNFs’ and CNTs’ bridging role in promoting the MPs of cementitious materials [250]. The rapid consumption of Ca(OH)_2_ produced during cement hydration, particularly at early ages, which is linked to the high reactivity of NS and NPs, is responsible for the increased compressive strength of concretes improved with NS. As a result, cement hydration is accelerated, resulting in more hydration products. NS particles can also enhance the blended cement’s particle packing density, resulting in a lower volume of larger pores in the cement paste, as shown in Figure 21 [251]. NC-containing mortar causes the yield stress to build up at the bottom layer before the subsequent layers are applied. As a result, the interface between layers becomes weak. Another study found that NC addition to 3DPC had more micropores in the interface than the control concrete due to its increased thixotropic characteristics (Figure 22) [252].

## 8. Conclusions and Future Recommendations

### 8.1. Conclusions

The authors assumed that nanoparticles (NPs) would have a brighter future in the fields of cement-based materials, alkali-activated fly ash (FA)-based materials, and alkali activation of other materials. NPs may become more common and widely used in the coming years once their high manufacturing costs are lowered. The impact of various nanomaterials (NMs) on the fresh state’s properties, the hardened state, the durability, 3D printing, and the microstructure of cementitious materials was reviewed considering more than 230 articles from the literature. Workability and rheological properties, compressive, tensile, and flexural strength, shrinkage, pore structure, porosity, durability, 3D printing materials, and microstructural analysis are all a part of the critical analysis used to alter the characteristics of the composite with various NMs. The following main conclusions are drawn from the comprehensive literature review, and study focus areas can be established.

Rheology is a valuable predictor for determining the flow behavior of cementitious materials to predict their performance in the fresh state in practical engineering applications. Such parameters are very likely to affect the properties of hardened concrete. By increasing the hydration rate and enhancing the microstructure of hydrated materials, NMs’ addition has a promising effect on both fresh and hardened properties of cementitious materials. Mechanical properties (MPs) of NMs have been studied extensively in the literature under various conditions. Due to the addition of NPs in cementitious materials, MPs are enhanced and support the use of NPs. Nano-silica (NS) has excellent characteristics, including a high melting point, high hardness, and chemical resistance. It is a promising material to enhance a cement-based material’s performance. Increased compression and flexural strength in the cement mortar can be achieved by adding NS. With the addition of 2% NS in high-volume fly ash (HVFA) concrete, the compressive strength increased considerably in the early days. The addition of NMs to hybrid fiber-reinforced cement composites positively affected the flexural and tensile strengths. The addition of nano-Al_2_O_3_ has no effect on early age cement paste’s compressive strength. NS and SF show the most impact on the compressive strength of HSC with the subsequent enhancements of 14% and 41% at a weight percentage of 2% and 12%, respectively. Additionally, a significant improvement in tensile strength was observed using 3% NS and 10% SF with an increase of 16% and 28%, respectively. The 3DPC containing NS and NC had significant improvements at an early age in its compressive, flexural, and interlayer bond strengths.

The autogenous shrinkage of cement-based materials containing NMs with increasing age follows the same pattern as that of reference samples. Alternatively, the addition of NS has a positive effect on drying shrinkage. The volume stability of magnesium phosphate cement (MPC) specimens is influenced by the addition of FA, SF, and ultra-fine FA. The addition of dipotassium hydrogen phosphate is an essential factor in MPC mortars. One of the hydration products in MPC mortar may be due to the presence of crystalline potassium chloride, which is formed by neutralizing potassium hydroxide. There appears to be a link between the expansion values and the MPC mortar’s potassium chloride content. The dipotassium hydrogen-phosphate-modified MPC mortar has numerous microcracks. In general, the addition of NMs to cementitious composites improves their durability by lowering the permeability of composites. The pore filling effect, pozzolanic nature, and NM reactivity are all factors that contribute to the reduction in the permeability of cementitious composites. However, it is important to evaluate the optimal content of NMs to have a positive effect. Beyond this, a higher content of NMs can have a negative impact on the performance of cementitious composites.

Material science lacks a unique standard for the most important foundations in the development of 3D structural printing. Structural materials with smart functionalities, such as self-sensing, self-assembly, and strain hardening, will advance the mechanized construction industry, allowing for the cost-effective manufacturing of energy-efficient buildings on the Earth as well as the implementation of 3D structural printing. Furthermore, green concrete technology has the ability to produce buildings that are more environmentally friendly. However, the feasibility of various aspects of using 3D structural printing technology to supply green concrete as the main feed to large-scale printers needs to be investigated. Developing cost-effective mega-scale printer devices with corrosion-resistant pumps capable of withstanding the alkaline nature of cement paste remains a challenge; despite advancements, the printing techniques required for 3D structural printing remain to be developed. The high cost of repair and maintenance and the economic issues currently prevent realistic 3D structural printing technology applications from being realized. The most well-known challenges are complicated fresh properties, appropriate MPs for structural applications, the use of reinforcements during the printing process, controlling autogenous shrinkage of printed components, and the brittleness of large-scale printed structures. Despite detailed assessments of the fresh properties of printable composites improved with NMs, knowledge of the 3D printable composite’s hardened properties is minimal. While some data on the MPs of cementitious composites are already available, information on nano-modified mixtures’ durability is severely restricted. The optimum dosage of NMs is a critical issue when designing a 3DPC mixture. An excessive NS or nano-clay (NC) dosage can cause an excessive increase in the specimen’s thixotropy, making extrusion difficult and resulting in a poor interface between layers. As a result, increased porosity and a decrease in strength could occur.

Furthermore, adding SF or FA to the mix has little effect on microstructural cracking. When 20% ultra-fine FA was added to MPC mortar, the number of microcracks was significantly reduced. As a result, ultra-fine FA is an ideal mineral admixture for improving the MPC mortar’s volume stability by reducing the amount of crystalline potassium chloride and microcracks. Fe_2_O_3_ powder may improve microstructural crystallization, react in the MPC system, create new hydration products, and has an excellent pore-filling effect, forming a denser microstructure according to scanning electron microscopy (SEM) and thermo-gravimetric analysis (T-G-A) analyses. An iron phosphate system analysis revealed that a higher curing temperature could result in a more acidic solution, promoting the reaction between Fe_2_O_3_ and ADP, resulting in a modified microstructure and new hydrates with good gel binding properties. The activation of Fe_2_O_3_ in the MPC system was confirmed. With the addition of nano-Al_2_O_3_, the agglomeration of particles resulted in the formation of a much denser microstructure. The SEM tests result support autogenous shrinkage and electrochemical testing findings, indicating that cement-based materials with NMs, CNTs, or NC have a more compact microstructure and more plentiful hydration products than the reference samples. The results of SEM analysis and mercury intrusion porosimetry (MIP) confirm a dense microstructure of the NS paste. NS results in a more compact paste with a significant reduction in pores between 0.1 and 10 µm in size. NS paste’s cumulative pore volume is approximately 25% lower than that of cement paste. There is a significant reduction in the cumulative pore volumes in high-volume fly ash (HVFA) pastes with a 2% NS addition, suggesting that the presence of NS is beneficial to pore alteration. The results show that NS has a significant impact on the total number of capillary pores and the pore diameter in HVFA pastes. SEM micrographs of a hybrid fiber-reinforced nanocomposite containing 1% NC show that it outperforms other specimens regarding hydration products on the fiber’s surface, suggesting a better fiber/matrix interface.

### 8.2. Future Recommendations

More research into the rheology of novel materials, such as highly pumpable concrete and ultra-high performance concrete, is required. Furthermore, it is essential to investigate the basic characteristics of printed components in order to determine whether additive manufacturing (AM) technology can be used for onsite construction. Since most studies on the effect of NMs have concentrated on permeability properties, it is suggested that future research look into the effect of NMs on cementitious composites’ resistance to physical attacks such as weathering and elevated temperatures. To determine the shrinkage behavior, microstructure improvements, characteristics of permeability, and toughness properties of concrete, more exploration is required of nano-integrated fiber-reinforced cementitious composites. Future research should consider improving the dispersion of higher concentrations of NMs in cement paste to increase the inhibitory effect on autogenous shrinkage. The impact of adding various NMs to 3D-printed concrete on thixotropy and early structural build-up could be investigated further. However, there are many demands and aspirations for the digital construction and building industry. Three-dimensional (3D) printing technology is being used in several ways, and new materials with added functions for 3D structural printing are being produced. Nonetheless, fabrication techniques are expected to move in two directions in the future: onsite fabrication of large elements and fabrication of high-resolution elements in a controlled environment. As a result, further research is required to fully comprehend NP effects on the engineering properties of 3D-printable materials. However, many areas need to be explored concerning the molding mechanism and strengthening NMs’ microstructure phase. NMs’ unique properties give them a wide variety of applications and great potential to be used in cementitious composites.

## Figures and Tables

**Table 1:** Effect of NMs on characteristics of the cementitious composite [16].

References	Type	Effect
Peyvandi, et al. [17]	Carbon Nanotubes (CNTs)	Reduction in permeability and resistivity to an aggressive environment
Pera, et al. [18]	Nano calcium carbonate (NCC)	Refinement of pores and quicker hydration reaction
Behnood and Ziari [19]	Nano-silica (NS)	Improved durability and less permeable
Hashimoto, et al. [20]	Nano clay (NC)	Higher pore refinement and thermal properties
Hashimoto, et al. [20]	Nano titanium (NTI)	Higher photo-catalytic activity, self-disinfection, and self-cleaning
Farzadnia, et al. [21]	Nano alumina (NA)	Minimum permeability and pore refinement
Han, et al. [22]	Nano magnetite (NMT)	Better mechanical properties, self-sensing, refinement of pores
Diab, et al. [23]	Nano metakaolin (NMK)	Lowering the permeability

**Table 2:** Physical properties and the optimal percentage of nanomaterials as retrieved from the literature [10].

Reference	Wang, et al. [57]	Bentz and Turpin [58]	Nili and Ehsani [59]	Salvador, et al. [60]	Nili and Ehsani [59]	Chithra, et al. [61]	Hou, et al. [62]
Details of the NM’s	NS	NCS	NA	NC	N-Fe_2_O_3_	N-Fe_3_O_4_	N-M_g_O
State	Powder	Liquid	Powder	Powder	Powder	Powder	Powder
Range of the average size of particles (nm)	15	8.5–9.0	12–18	3	13–17	100	100
Color	White	White	White	Pale White	Brown	Dark Brown	White
Density (gram/cm^3^)	2.2–2.6	1.21	0.1	2.29	0.15	4.8	3.58
Formula (chemical)	SiO_2_	SiO_2_	Al_2_O_3_	Al_2_Si 2O_5_ (O-H)_4_	Fe_2_O_3_	Fe_3_O_4_	M_g_O
Optimized %age	3	3	1	1.9	1	2	7.5

**Table 3:** The influence of NS on the strength of cement pastes [124].

Nano Content (%)	0 and 3	0, 1, 2, and 3	0, 1, 2, 3, and 5	0, 0.5, 1, 2, and 5	0, 0.2, 0.4, 0.6, and 0.8	0, 0.8, and 3.8	0 and 0.6	0 and 0.5	0, 2, 5, and 10	0, 2, 4, 6, and 10
Effect	It improved compressive and flexural strength. The optimum content is 3% by weight.	Increased compressive strength. The optimum content is 2%, followed by 1%, by weight.	Enhanced compressive and bond strength. Optimum content is 5%, followed by 3%, by mass.	Better compressive strength. The optimum content is 0.5%, followed by 2%, by weight.	Higher compressive strength. The optimum content is 0.6%, followed by 0.4%, by weight of cement.	Increase in the compressive strength.	Improvement in compressive strength.	Enhancement in the strengths.	Increase in the indirect tensile strength. The optimum content is 2% by weight of cement.	Increment in the compressive strength. The optimum content is 10%, followed by 6%, by the weight of the cement.
Authors	Qing, et al. [125]	Kuo, et al. [36]	Qing, et al. [126]	Stefanidou and Papayianni [127]	Shih, et al. [82]	Berra, et al. [128]	Gaitero, et al. [129]	Pourjavadi, et al. [130]	Shebl, et al. [131]	Thuadaij and Nuntiya [132]

**Table 4:** Structural parameters of pores in various samples [216].

Sample	Plain Cement	GO Cement	NS Cement	Hybrid Cement
MIV (mL/g)	0.220	0.207	0.196	0.171
Average diameter of pore (nm)	19.5	18	17.1	16.2
Porosity (%)	32.38	32.04	30.01	24.12
Surface to volume ratio (mL/g)	46.374	44.475	44.233	42.880

**Table 5:** Inclusion of nano clay, graphene-based materials, and nano-silica in printable mixes [219].

Type of NM	NC	NS	Graphene-Based Materials
Specification	Highly purified attapulgite NC	-	Nano graphite platelets (NGPs)
	Highly purified magnesium alumino-silicate clay	-	
	Nano attapulgite clay	-	
	Hydrophilic bentonite NC	-	
Quantities	0-0.1-0.3-0.5% by mass of binder (MB)	0.5%–1% by mass of solid	0.1%–0.5% by MB
	0%–3% by mass of cement (MS)	0.5-2-3.5% by MS	
	0.1%–0.5% by MB	1, 2, 3% by MS	
	0.5%–1% by mass of solid	-	
Optimum	0.5% by MB	-	1.0% by MB
	0.5%–1% by MS	3.5% by MS	
	0.5% by MB	1% by MS	
Effect	Boost in cohesion and static yield strength	Boost in the velocity of the structural build-up of the paste	Effective rheology modification agent with increased mechanical performance
	Facilitates re-flocculation and improves the thixotropic properties of the mixture	Decreased plastic shrinkage	
	Boosts static yield stress without affecting apparent viscosity	Boost in the re-flocculation rate	
	Thickening effect, increment in the static yield strength	-	
References	Qian, et al. [242], Quanji, et al. [101], Panda, et al. [104], Reales, et al. [240]	Reales, et al. [240], Sonebi, et al. [243], Kruger, et al. [244]	Chougan, et al. [238]

## Data Availability

Data sharing not applicable.
